# Predictability of gene ontology slim-terms from primary structure information in *Embryophyta* plant proteins

**DOI:** 10.1186/1471-2105-14-68

**Published:** 2013-02-26

**Authors:** Jorge Alberto Jaramillo-Garzón, Joan Josep Gallardo-Chacón, César Germán Castellanos-Domínguez, Alexandre Perera-Lluna

**Affiliations:** 1 Departamento de Ingeniería Eléctrica, Electrónica y Computación, Universidad Nacional de Colombia sede Manizales, Campus La Nubia, km 7 vía al Magdalena, Manizales (Caldas), Colombia; 2 Centre de Recerça en Enginyeria Biomèdica, ESAII, Universitat Politècnica de Catalunya, Pau Gargallo 5, 08028 Barcelona, España; 3 Centro de Investigación, Instituto Tecnológico Metropolitano, Calle 73 No 76A - 354, Medellín (Antioquia), Colombia; 4 Centro de Investigación Biomédica en Red en Bioingeniería, , Biomateriales y Nanomedicina (CIBER-BBN), España; 5 Planta de Tecnologia dels Aliments, Universidad Autónoma de Barcelona, 08193 Cerdanyola del Vallès, Catalonia, España

## Abstract

**Background:**

Proteins are the key elements on the path from genetic information to the development of life. The roles played by the different proteins are difficult to uncover experimentally as this process involves complex procedures such as genetic modifications, injection of fluorescent proteins, gene knock-out methods and others. The knowledge learned from each protein is usually annotated in databases through different methods such as the proposed by The Gene Ontology (GO) consortium. Different methods have been proposed in order to predict GO terms from primary structure information, but very few are available for large-scale functional annotation of plants, and reported success rates are much less than the reported by other non-plant predictors. This paper explores the predictability of GO annotations on proteins belonging to the *Embryophyta* group from a set of features extracted solely from their primary amino acid sequence.

**Results:**

High predictability of several GO terms was found for Molecular Function and Cellular Component. As expected, a lower degree of predictability was found on Biological Process ontology annotations, although a few biological processes were easily predicted. Proteins related to transport and transcription were particularly well predicted from primary structure information. The most discriminant features for prediction were those related to electric charges of the amino-acid sequence and hydropathicity derived features.

**Conclusions:**

An analysis of GO-slim terms predictability in plants was carried out, in order to determine single categories or groups of functions that are most related with primary structure information. For each highly predictable GO term, the responsible features of such successfulness were identified and discussed. In addition to most published studies, focused on few categories or single ontologies, results in this paper comprise a complete landscape of GO predictability from primary structure encompassing 75 GO terms at molecular, cellular and phenotypical level. Thus, it provides a valuable guide for researchers interested on further advances in protein function prediction on *Embryophyta* plants.

## Background

The universe of protein functions can be summarized through the use of the Gene Ontology (GO) project, which aimed to construct controlled and structured vocabularies known as ontologies, and apply them in the annotation of gene products in biological databases [1]. *Molecular Function* ontology refers to biochemical activities at the molecular level, no matter what entities accomplish that function or the context where it takes place; *Cellular Component* ontology refers to the specific sub-cellular location where a gene product is active, describing different parts of the eukaryotic cell; *Biological Process* ontology refers to a series of events with a defined beginning and end, to which the gene product contributes. Currently, as of February 2013 there are 38137 defined GO terms, distributed over 9467 molecular functions, 3050 cellular components and 23928 biological processes. However, in spite of such variety of functions, all proteins share a common basic configuration: a linear polypeptide chain composed by different combinations and repetitions of the twenty amino acids encoded by genes. Although, currently there are almost 8 million sequences in non-redundant databases, for most, we know just that amino acid sequence deduced from the DNA chain [2]. Assessment of protein functions requires, in most cases, experimental approaches carried out in the lab. Unfortunately, these procedures must be focused on specific proteins or functions, and require either cloned DNA or protein samples from the genes of interest. Additionally, the function of many proteins may be related to its own native environment. Such perspective has led some authors to conclude that the only effective route towards the elucidation of the function of some proteins may be computational analysis and prediction from amino acid sequences that later can be subjected to experimental verification [3].

Many approaches have been developed in this matter (for complete revisions, see [4-6]). One of the earliest applications, yet still one of the more popular bioinformatics tools is the Basic Local Alignment Search Tool for proteins (BLASTP) [7] which has been applied for obtaining annotation transfers based on sequence alignments. Also, a high number of methods (GOblet [8], OntoBlast [9], GOFigure [10] and GOtcha [11]) are based on the idea of refining and improving initial results from classic alignment tools such as BLASTP, by performing mappings and weightings of GO terms associated to BLASTP predictions. However, in such methods, the failure of conventional alignment tools to adequately identify homologous proteins at significant E-values is not considered [12]. The same applies for some more recent methods that have improved specific points of this methodology such as speeding up the procedure through decision rules [13], including additional functionality for visualization and data mining [14] or also including some statistics of GO terms to refine selection [15]. In order to avoid the dependency to BLAST alignments in the cases where the alignment-based annotation transfer approach is not so effective, more recent methods have used machine learning techniques trained over feature spaces of physical-chemical, statistical or locally-based attributes. Those methods employ techniques such as neural networks (ProtFun [16]), Bayesian multi-label classifiers [17] and support vector machines (SVM-Prot [18], GOKey [19], PoGO [20]), obtaining high performance results in their own respective databases, mostly composed by model organisms such as bacteria and a few high order species.

There are, however, several aspects that must be discussed about current performances in prediction of GO terms, when applied to non-model organisms such as land plants (*Embryophyta*). First, from the previously described methods, only Blast2GO [14] was specialized for predicting GO terms in plant proteins. In fact, as it is pointed out by the authors of Blast2GO, very few resources are available for large-scale functional annotation of non-model species. Some methods specialized on vegetative species have been proposed recently, but they are only intended for performing cellular component predictions (Predotar [21], TargetP [22], Plant-mPloc [23]). Moreover, Predotar and TargetP can discriminate among only three or four cellular location sites. Plant-mPloc, in turn, covers twelve different location sites and it was rigorously tested over a set of proteins with less than 25% of identity among them, where homologue-based tools like BLASTP would certainly fail. For such dataset, they obtained an overall success rate of 63.7%, much less than reported by other cellular location predictors tested over non-plant datasets. Second, none of the existing methods can be used to deal with plant proteins that can simultaneously exist or move between two or more different location sites [23], or belong to multiple functional classes at the same time [24].

In order to improve the performance of current GO term predictors for land plants, it would be useful to have a better understanding of the underlying relationships between primary structure information and protein functionality. However, the structure of the machine learning models behind high-accuracy predictors often makes difficult to understand why a particular prediction was made [24]. In this sense, a recent method called Yloc [24] was proposed for analyzing what specific features are responsible for given predictions. This method, nevertheless, is not intended to predict GO terms, but instead, it uses annotation information from PROSITE [25] and GO as inputs to the predictor. Additionally, their study is only focused on predicting protein locations in the cell.

Since most of the current GO prediction methods are limited to a few arbitrary functional classes and single ontologies, they cannot provide any information about relationships on the predictability at the various levels of protein functionality (molecular, cellular, biological), which could be another key element for determining how the information of the primary structure is related to protein function.

This work presents an analysis on the predictability of GO terms over the *Embryophyta* group of organisms, which is composed by the most familiar group of plants including trees, flowers, ferns, mosses, and various other green land plants. The analysis provides the following key elements: predictions are made by using features extracted solely from primary structure information; analysis comprises most of the available organisms belonging to the *Embryophyta* group; biases due to protein families are avoided by considering only proteins with low similarity among them and a strong evidence of existence; predictions and analysis are made over a set of categories belonging to the three ontologies; proteins are allowed to be associated to several GO terms simultaneously.

Results from this work answer whether it is possible to predict most GO-slim terms from primary structure information, what categories are more susceptible to be predicted, which ontology is most related to the information contained in the primary structure and what relationships among ontologies could be influencing the predictability at different levels of protein functionality in land plants.

## Methods

The implemented methodology for assessing predictability of GO terms in *Embryophyta* proteins comprises the following parts: (i) selection of the protein sequences conforming the database in order to cover the highest number of available plant proteins, while ensuring high confidence annotations and avoiding possible biases; (ii) categories describing positive and negative samples associated to each GO term are determined in order to minimize the impact of hierarchical relationships; (ii) protein sequences are mapped into feature vectors that encode a number of attributes of varied nature; (iii) computed features are clustered into groups of similar information content; (iv) one binary classifier is learned for each GO term and each feature cluster in order to evaluate the prediction power of individual clusters, and finally (v) one binary classifier is learned for each GO term using the whole set of features in conjunction with an automatic feature selection strategy in order to determine the global predictability of each GO term.

The following subsections describe the methods employed for each part of the methodology. All simulations were implemented on the *R* environment for statistical computing [26]. Additional tools were mainly provided by Bioconductor [27], and the *seqinR* package [28], all of them freely distributed under the *GNU* General Public License.

### Database

The database comprises all the available *Embryophyta* proteins at UniProtKB/Swiss-Prot database ( [29], file version: 10/01/2013), with at least one annotation in the Gene Ontology Annotation (GOA) project ( [30], file version: 7/01/2013). The resulting set comprises proteins from 189 different land plants.

In order to avoid the presence of protein families that could bias the results, the dataset was filtered at several levels of sequence identity using the Cd-Hit software [31]. The main results are reported for the lowest identity cutoff (30%). However, additional analyses at 40%, 50%, 60%, 70% and 80% were also performed in order to provide further information on the robustness of the method.

The main set comprises a total of 3368 protein sequences, from which 1973 sequences are annotated with molecular functions, 2210 with cellular components and 2798 with biological processes. Automatically-assigned annotations were not included in the analyses.

### Definition of classes

Although, in principle, the method can be trained to predict any GO term for which there are enough training sequences, all tests were performed over the set of categories defined by the plants GO slim developed by The Arabidopsis Information Resource - TAIR ( [32], file version: 14/03/2012). This choice was made because GO includes a large number of categories that do not occur in plants, due to its broad size. In turn, slims are smaller, more-manageable sub-sets of GO, that focus on terms relevant to a specific problem or data set [33], thus allowing to generate higher-level annotation more robust to tests of statistical significance [34].

Positive and negative samples associated to each GO term are selected by considering the propagation principle of GO. If a protein is predicted to be associated to any given GO term, it must be automatically associated to all the ancestors of that category and thus, it is enough to predict only the lowest level entries. Consequently, for each GO term, positive samples are all those proteins that have been annotated with this term or any of its descendants, excepting those descendants that are also included as categories. All the remaining samples in the database are selected as negative samples for that GO term. In order to explicitly note that some GO terms are not including their descendants categories, such “incomplete” GO terms are marked with an asterisk throughout the paper.

After defining the membership of the sequences, categories with less than 30 proteins were discarded because they did not have enough samples to train a statistically reliable classifier. The final set is thus comprised by 14 GO terms in the molecular function ontology, 20 GO terms in the cellular component ontology and 41 GO terms in the biological process ontology. Table 1 shows the final list of categories, as well as the acronyms used to cite them throughout this paper and the number of samples in each one for the 30% identity cutoff.

**Table 1:** Definition and size of the classes

**Class**	**Acronym**	**Size**	**Class**	**Acronym**	**Size**
**Molecular Function**			**Biological Process**		
Nucleotide binding	Ntbind	47	Reproduction*	Reprod*	337
Molecular function*	MF*	268	Carbohydrate metabolic process	ChMet	315
DNA binding	DnaBind	107	Generation of precursor metabolites	MetEn	150
Transcription factor activity	TranscFact	307	and energy		
RNA binding	RnaBind	43	Nucleobase, nucleoside, nucleotide,	NaMet*	712
Catalytic activity*	Catal*	334	nucleic acid metabolic process*		
Receptor binding	RecBind	38	DNA metabolic process	DnaMet	191
Transporter activity	Transp	125	Translation	Transl	82
Binding*	Bind*	173	Protein modification process	ProtMod	391
Protein binding*	ProtBind*	630	Lipid metabolic process	LipMet	324
Kinase activity	Kinase	68	Transport	Transport	531
Transferase activity*	Transf*	173	Response to stress	StressResp	790
Hydrolase activity	Hydrol	190	Cell cycle	CellCycle	234
Enzyme regulator activity	EnzReg	41	Cell communication*	CellComm*	66
			Signal transduction	SigTransd	305
			Cell-cell signaling	Cell-cell	53
**Cellular Component**			Multicellular organismal development*	MultDev*	490
Cellular component*	CC*	234	Biological process*	BP*	879
Extracellular region	ExtcellReg	109	Metabolic process*	Met*	279
Cell wall	CellWall	77	Cell death	CellDeath	95
Intracellular*	Intracell*	167	Catabolic process	Catabolic	479
Nucleus*	Nucleus*	421	Biosynthetic process*	Biosint*	1125
Nucleoplasm	NuclPlasm	51	Response to external stimulus*	ExtResp*	65
Nucleolus	Nucleolus	84	Tropism	Tropism	36
Cytoplasm*	CitPlasm*	168	Response to biotic stimulus	BioResp	275
Mitochondrion	Mitochond	244	Response to abiotic stimulus	AbioResp	642
Endosome	Endosome	58	Anatomical structure morphogenesis	StrMorph	366
Vacuole	Vacuole	171	Response to endogenous stimulus	EndoResp	332
Peroxisome	Peroxisome	32	Embryonic development	EmbDev	139
Endoplasmatic reticulum	EndRet	109	Post-embryonic development*	PostDev*	375
Golgi apparatus	GolgiApp	100	Pollination	Poll	43
Cytosol	Cytosol	389	Flower development	FlowerDev	228
Ribosome	Ribosome	98	Cellular process*	CP*	1486
Plasma membrane	PlasmMb	353	Response to extracellular stimulus	ExtcellResp	59
Plastid	Plastid	696	Photosyntesis	Photosyn	102
Thylakoid	Thylk	147	Cellular component organization	CellOrg	757
Membrane*	Mb*	472	Cell growth	CellGrowth	133
			Protein metabolic process*	ProtMet*	187
			Cellular homeostasis	CellHom	53
			Secondary metabolic process	SecMet	164
			Cell differentiation	CellDiff	267
			Growth*	Growth*	64
			Regulation of gene expression,	RGE	103
			epigenetic		

The list of GO terms covered by this analysis is intended to provide a complete landscape of GO predictability at the three levels of protein functionality in *Embryophyta* plants. For classification purposes, classes marked with an asterisk (*) were redefined. The number of samples in those categories corresponds to the sequences associated to that class and none of its also listed descendants.

### Characterization of protein sequences

Protein sequences are mapped into feature vectors by extracting three types of attributes: physical-chemical features, primary structure composition statistics and secondary structure composition statistics (see Table 2). The first group provides information directly related with biochemistry of the molecule: weight, polarity of amino acid side chains, isoelectric point, and hydropaticity index (GRAVY). The second group is based on counting the occurrences of all possible subsequences of a fixed length *n*. Only features corresponding to *n*={1,2} are employed, provided that the size of the feature space grows exponentially with *n*, and simple counts were converted into relative frequencies (summing to one). The third group is analogous to the second one, but applying such characterization to the predicted secondary structure of the protein. Predictions were computed with the Predator 2.1 software [35], whose output is a linear sequence with three structural domains: alpha helices, beta sheets and coils.

**Table 2:** Initial set of features extracted from amino acid sequences

**Nature**	**Description**	**Number**
Physical-chemical	Sequence length	1
	Molecular weight	1
	Positively charged residues (%)	1
	Negatively charged residues (%)	1
	Isoelectric point	1
	GRAVY	1
Primary structurestatistics	Amino acid frequencies	20
	Amino acid dimer frequencies	400
Secondary structurestatistics	Structure frequencies	3
	Structural dimer frequencies	9
	Total	**438**

Features are divided into three broad categories: physical-chemical features, primary structure composition statistics and secondary structure composition statistics.

In the case of ambiguous characters in the amino acid sequence, each feature was computed as its statistical expected value, with natural abundance percentages of amino acids as their prior probabilities. Additionally, since different groups of features are very heterogeneously scaled, *z-score* normalization was performed so that each feature has a zero mean and unitary standard deviation.

The full feature matrix is provided in the supplementary material along with a file specifying the membership of samples to each category.

### Feature clusters

As a first step to perform an analysis of discriminant features for each GO term, features were hierarchically clustered into groups of similar information content (Additional file 1). For this purpose, the Ward clustering algorithm was used, with absolute Pearson correlation distance as metric. This procedure yielded 15 clusters that are summarized in Table 3 (a complete description can be found in the Additional file 2) and are used for assessing the influence of specific feature groups on the predictability of each category.

**Table 3:** Feature clusters

**Group**	**Main feature**	**Size**	**Group**	**Main feature**	**Size**
1	Protein length	34	9	Proline	14
2	Negative charge /Acidic	8	10	Glutamine	35
3	Positive charge /Basic	30	11	Arginine	26
4	Alanine	10	12	Tryptophan	38
5	Cysteine	38	13	Tyrosine	35
6	Hidrophobic	46	14	Alpha helices	6
7	Histidine	29	15	Beta sheets	4
8	Asparagine /Methionine	85			

Description of the clusters of features with similar information content. A complete description of features belonging to each cluster can be found in the Additional file 2.

### Feature selection strategy

The feature selection procedure is carried out in the second part of the Results and discussion section, where the global predictability of each GO term is evaluated by using the whole feature set. Since redundant features would possibly overfit the training data, an analysis of relevance and redundancy was applied. Let **f**_*i*_, *i*=1,2,…,*n*, be the initial set of features, **y** be the vector of labels, *c*_*ij*_=cor(**f**_*i*_,**f**_*j*_) be the linear correlation computed between any pair **f**_*i*_ and **f**_*j*_ and *c*_*iy*_=cor(**f**_*i*_,**y**) be the linear correlation between **f**_*i*_ and **y**. Defining this, relevance of features can be quantified by computing *c*_*iy*_ for all features and then, redundant ones can be identified by analyzing the *n*×*n* feature correlation matrix. In order to speed up the calculations, an algorithm based on the *Fast Correlation-Based Filter* of [36] was used.

### Decision making

In order to allow samples to be associated to multiple categories, decision making was implemented following the one-against-all strategy. The method produced a strong class imbalance since it trains a number of binary classifiers, each one intended to recognize samples from one class out of the whole training set. To overcome the problems that imbalanced classes commonly produce in pattern recognition techniques, the Synthetic Minority Over-sampling Technique (SMOTE) was employed [37].

A support vector machine (SVM) with Gaussian kernel was used for running all the classification tests. This SVM is trained with the ’kernlab’ package, available in R-CRAN [38]. Dispersion of the kernel and trade-off penalization parameter of the SVM are tuned for each test with a particle swarm optimization meta-heuristic, a bio-inspired optimization method that has been used in multiple applications in the past years [39].

In order to estimate the performance of the predictive model, a 5-fold cross-validation strategy is implemented. In such strategy, the test procedure is repeated five times, and each time an 80% of the data is used for adjusting the SVM parameters and training the model, while the remaining 20% is used as testing samples. This strategy also allows providing an estimation of the reliability of the model by computing the variability of the results through the five repetitions.

## Results and discussion

### Analysis of predictability with individual feature clusters

**Figure 1:**
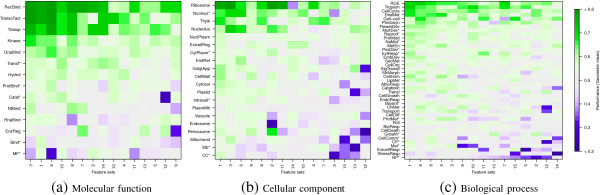
**Prediction performance with different feature clusters for the three ontologies (a) Molecular function, (b) Cellular component and (c) Biological process.** Rows represent classes in Table 1 while columns represent feature groups in Table 3. For each ontology, best predicted categories are ordered from top to bottom while most discriminant feature groups are ordered from left to right.

Classification results with individual feature clusters, for an identity cutoff of 30%, are condensed in Figure 1. The square root of the product between sensitivity and specificity (geometric mean), is depicted as global performance measure and the color scale has been adjusted to highlight the highest (green) and the lowest (blue) performances. Note that the rows and columns have been ordered to explicitly locate best predicted GO terms on top and most discriminant groups to the left.

Figure 1(a) shows the analysis for the molecular function ontology. For all feature groups, *Receptor binding* achieved the highest classification scores. This category is intended to comprise proteins that interact selectively and non-covalently with one or more specific sites on a receptor molecule. About 63% of the proteins associated to this category in the database are proteins involved with binding of serine/threonine kinase receptors, which turned out to be easily predicted from most of the defined features.

*Transcription factor activity* achieved was easily predicted from the feature groups 1, 3, 6, 8 and 14. Not so surprising is the fact that *DNA binding* also presents a similar behavior since most transcription factors must interact with DNA molecules and consequently they are also included in this category. However it is worthy to note that several other proteins also perform DNA cleavage, such as polymerases, nucleases and histones, and they were also well predicted from the same feature groups. The conclusion from these results becomes more evident by observing the results of the *DNA metabolic process* in Figure 1(c), which confirm the high predictability of all proteins involved with transcription when using the mentioned features groups. A similar behavior is also observed for nucleus* in Figure 1(b), supported by the fact that the transcription process is mostly carried out in that sub-cellular location.

*Transporter activity* refers to proteins that enable the directed movement of substances into, out of, within or between cells. Most of them are integral transmembrane proteins, that are distinguished by their high content of hydrophobic residues [40]. In fact, some of the highest performances of *transporter activity* were reached with the groups 3 and 6, which include GRAVY index as well as monomer and dimer frequencies of three out of the four most hydrophobic residues: leucine, isoleucine and phenylalanine. Additionally, predictability of this molecular function is reflected, while in a minor degree, on the *transport* biological process, which reaches its highest values for the same feature groups (see Figure 1(c)). The main difference between those GO terms lies in that *transport* is a broader category, including external agents such as oxygen carriers and lipoproteins that perform transport within multicellular organisms.

On the other hand, the root node of the molecular function ontology was GO terms with the lowest average prediction performances. Remember that the root node contains the proteins that do not belong to any of its descendant categories, so it keeps a small set of sequences of a sparse nature, which explains the impossibility to model and predict them as a group. It is interesting to note that the same behavior is observer for the other two ontologies (Figures 1(b) and 1(c)).

Concerning the cellular component ontology, it can be observed in Figure 1(b) that *ribosome* category has reached the highest classification accuracies, specially with groups 1, 2, 3 and 11. Such groups mainly consist of the four charged residues: lysine, arginine, glutamic acid and aspartic acid. This can be explained since ribosomal proteins must interact with the negatively charged phosphodiester bonds in the RNA backbone, so they are expected to have a high percentage of positively charged residues to neutralize such charge repulsion. In agreement with this, [40] describes the composition of isolated ribosomal proteins as showing a high percentage of lysine and arginine residues and a low aromatic content. Hence, there is enough evidence to establish that ribosomal proteins are another highly predictable category from primary structure information.

As explained before, nucleus* becomes easily predicted from the same feature groups that have shown high discriminant capabilities for transcription related proteins. A similar behavior is also observed for proteins belonging to the *nucleolus* component, which encompasses proteins including RNA polymerases, transcription factors, processing enzymes and ribosomal proteins among others, which must interact with nucleic acids and have shown low isoelectric points in comparison to the remaining proteins in the database.

*Thylakoid* proteins also presented high prediction performances with several feature groups. Further studies would be required to explain this results.

Broad categories such as membrane* showed poor performances with most feature groups, presumably due to its high diversity. However, some rather well-defined categories such as *mitochondrion* and *perixosome* were also ranked in the lowest places in Figure 1(b), simply proving to be poorly predictable from the extracted feature groups.

Concerning Figure 1(c), the biological process that was better predicted for most group features is *regulation of gene expression, epigenetic*. This GO term encloses proteins involved in modulating the frequency, rate or extent of gene expression and is highly composed by histones. In fact, since histones are highly alkaline proteins, it is consistent to observe that this category became particularly well predicted from groups 3, 6 and 7, which are mainly conformed by frequencies of phenylalanine, leucine, isoleucine, lysine and histidine residues. Also, cysteine related frequencies were highly discriminant for *regulation of gene expression, epigenetic* (group 5 which can be explained by the fact that altering the redox state of cysteines serves for modulating protein activity, and several transcription factors become activated by the oxidation of cysteines that form disulfide bonds [41].

*Tropism* and *Cell Cycle* also appeared near the top of Figure 1(c), just before *DNA metabolic process* which was already discussed.

### Analysis of predictability with the full set of features

**Figure 2:**
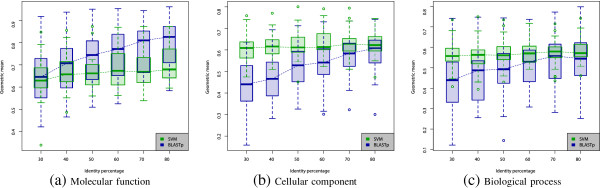
**Performance variation in function of the identity cutoff for the three ontologies (a) Molecular function, (b) Cellular component and (c) Biological process.** Green and blue plots show the variation of the general prediction performances for SVM and BLASTP, respectively, according to the identity percentage cutoff used in the dataset. Boxplots show the dispersion throughout the 75 GO terms.

Analyses in the previous section were done after discarding sequences with identities superior to 30%. Otherwise, the predictability of certain terms could be enhanced from the fact that many proteins in training and testing sets are copies (or close relatives) from another, rather than from predictive value of certain sequence-derived features. However, in order to provide further information on the robustness of the proposed methodology when the identity cutoff changes, Figure 2 presents an analysis of predictability with the full feature set (although applying the feature selection procedure described in the Methods section), while varying the identity cutoff. For comparison purposes, results achieved by BLASTP are depicted in blue, while results of the proposed methodology are depicted in green. The first thing that can be noted from Figure 2 is the fact that alignment-based predictions are more sensitive to the variation of the identity percentage than the proposed methodology. It can be clearly seen that BLASTP suffers a strong performance degradation as the identity filter is more stringent, while the performance of the proposed methodology remains more stable. Moreover, although in Figure 2(a) it can be seen that, when predicting molecular functions, BLASTP is superior than the proposed methodology for high identity cutoffs, the difference at 30% is not statistically significant. Conversely, the proposed methodology clearly outperforms BLASTP for low identity percentages when predicting cellular components and biological processes (Figures 2(b) and 2(c)).

**Figure 3:**
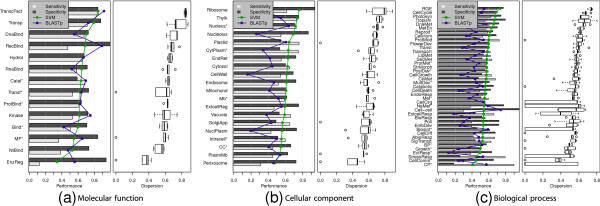
**Prediction performance with the complete set of features for the three ontologies (a) Molecular function, (b) Cellular component and (c) Biological process.** Bars in the left plots show sensitivity and specificity of SVMs. Lines depict geometric mean as a global performance measure for SVM (green) and BLASTP (blue). Right plots depicts variability throughout the five folds of cross-validation. For each ontology, best predicted categories are ordered from top to bottom.

Figure 3 depicts detailed results of predicting each class with the full feature set for an identity cutoff of 30%. Left plots show sensitivity, specificity and geometric mean (green line) achieved with the five-fold cross-validation procedure, while right plots depicts boxplots for analyzing performance variation throughout the five repetitions. Left plots also depicts the performance of the BLASTP algorithm for comparison purposes (blue line). Similar figures for the whole sweep of identity cutoffs are presented in the Additional file 3.

Note that GO terms were ordered again from top to bottom according to their predictability, but this order is not strictly the same as in Figure 1. Some interesting results in Figure 3(b) are provided by categories such as *plastid*, which was not easily predicted with any feature set independently, but reached medium to high classification results when the complete set was used. Such behavior is a clear example of the multivariate associations that could be missed when analyzing only individual feature sets.

Other results were consistent with the insights provided by the previous analyses, showing that some of the best predicted GO terms were *transporter activity*, *transcription factor activity*, and *DNA binding* in molecular functions; *ribosome*, nucleus*, *nucleolus* and *thylakoid* in cellular components; *regulation of gene expression, epigenetic*, *Cell cycle*, *Photosyntesis* and *DNA metabolic process* in biological processes.

A reduced number of categories had performances under 50%, most of them from the biological process ontology and a few form the molecular function ontology. It is important to note that the majority of those categories achieved very high specificities and low sensitivities, pointing out to a high dispersion of such categories over the feature space, which yields to a very high number of false negatives. Also, the high dispersions observed in the boxplots for some of the worst predicted classes demonstrate that there is a high variability among repetitions of the experiment which means that those low performances are not confident. Conversely, the categories with high performances show also low dispersions associated to them, hinting consistency in the predictors.

Although the main purpose of this work is not to design a highly accurate GO term predictor, but to provide a comprehensive analysis of the predictability of GO terms from primary structure information, it is important to mention how this method compares with currently used prediction tools. The blue and green lines in Figure 3 represent the prediction performances of BLASTP and the SVM based predictor used in this work, respectively. Both methods were tested over the same database described in the Methods section. From Figure 3(a) it is possible to conclude that the two methods provide similar prediction capabilities for the molecular function ontology at this identity cutoff. However, Figures 3(b) and 3(c) show that the SVM out-performed BLASTP for the cellular component and biological process ontologies, with only a few exceptions. It is also important to point out that the results achieved here are competitive with those reported by [23], which is one of the more recent and effective predictors dedicated to plant proteins.

**Figure 4:**
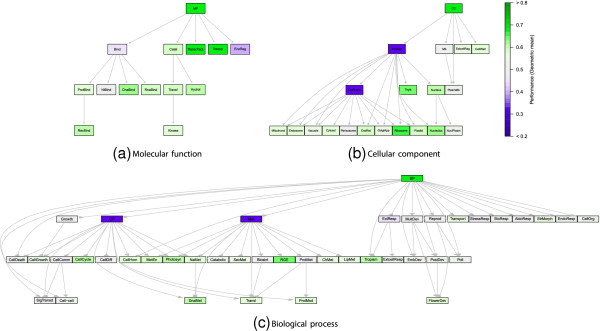
**Propagated prediction performance for the three ontologies (a) Molecular function, (b) Cellular component and (c) Biological process.** Prediction performance when propagating predictions of children nodes to their parents. Note that asterisks in the category names have been removed since categories include all their member now.

Finally, Figure 4 depicts the accuracy obtained in each category, when predictions of inferior GO terms were propagated up to their parents. Observe that asterisks have been removed to point out that GO terms are now including all their descendants.

It is notable how categories with the major number of descendants have been negatively affected by their false positives. This is especially observed in Figure 4(b) for *cytoplasm*, and *intracellular*, and Figure 3(c) for *cellular process* and *metabolic process*. Conversely, a few classes that were lacking sensitivity were favored by the contributions of their descendants, as it is the case of the root nodes of the ontologies.

## Conclusions

An analysis of GO terms predictability in land plants proteins was carried out in order to determine single categories or groups of related functions that are more related with primary structure information. For this purpose, pattern recognition techniques were employed over a feature set of physical-chemical and statistical attributes computed over the primary structure of the proteins. High predictability of several GO terms was observed in the three ontologies. Proteins associated to transport activities showed high correct prediction rates when using hydropathicity related features. Also, proteins involved with transcription (and therefore associated to the nucleus) presented high discriminability from the extracted features. Ribosomal and other proteins involved with translation, proved to be highly predictable from features related to electric charges of the amino acid sequence. At the biological process level, proteins related to regulation of gene expression and nucleic acid metabolic process were easily predicted, while some other biological processes showed low predictability from the extracted primary structure features. The information derived from this study could be used to get further improvement in prediction performances by combining the information from SVM classifiers with annotation transfer methods.

## Competing interests

The authors declare that they have no competing interests.

## Authors’ contributions

JAJG wrote the R scripts, designed and developed the workflow of the experiments and analyses. JJGC contributed to the biological analysis of the results. CGCD revised and approved the manuscript. APL suggested the main ideas and guided through the overall process. All authors contributed with the final manuscript.

## Supplementary Material

Additional file 1 **Data Set.** The main set comprises a total of 3368 protein sequences, from which 1973 sequences are annotated with molecular functions, 2210 with cellular components and 2798 with biological processes. Automatically-assigned annotations were not included in the analyses.
Two files are provided, both of them in character separated value (CSV) format; comma is used for the decimal point and a semicolon as column separator, according to the Excel convention for CSV files in most Western European locales.
**features.csv**: Contains the feature matrix of 3369 rows (3368 proteins + header) and 439 columns (438 features + header). The first column contains the Uniprot identifiers of proteins and the first row contains the feature names. Position (1,1) is an empty field.
**labels.csv**: Contains the membership matrix of 3369 rows (3368 proteins + header) and 76 columns (75 GO terms + header). The first column contains the Uniprot identifiers of proteins and the first row contains the GO keys of functional categories. Position (1,1) is an empty field. Each position in the matrix contains the number zero or the number one to establish whether or not the protein of that row is associated to the GO term corresponding to that column. Click here for file

Additional file 2 **Feature clusters description.** Table describes the feature clusters derived from correlation analysis. For this purpose, the Ward clustering algorithm was used, with absolute Pearson correlation distance as metric. A single letter stands for a single amino acid frequency according to their one-letter code, while letter pairs stand for dimmer frequencies. Also, symbols *α*, *β* and −, stand for frequencies of alpha helices, beta sheets and coils respectively. Click here for file

Additional file 3 **Detailed results for several identity thresholds.** Figures depicting detailed results for each class with the full feature set, for several identity cutoffs. Left plots show sensitivity, specificity and geometric mean (green line) achieved with the five-fold cross-validation procedure, while right plots depicts boxplots for analyzing performance variation throughout the five repetitions. Left plots also depicts the performance of the BLASTP algorithm for comparison purposes (blue line). Click here for file
