# Supplementary material for: Predictability of gene ontology slim-terms from primary structure information in Embryophyta plant proteins
Source: BMC Bioinformatics. 2013 Feb 26;14:68. doi: 10.1186/1471-2105-14-68 (PMC3660269; doi:10.1186/1471-2105-14-68)
Supplement: Additional file 2 — Feature clusters description. Table describes the feature clusters derived from correlation analysis. For this purpose, the Ward clustering algorithm was used, with absolute Pearson correlation distance as metric. A single letter stands for a single amino acid frequency according to their one-letter code, while letter pairs stand for dimmer frequencies. Also, symbols α, β and −, stand for frequencies of alpha helices, beta sheets and coils respectively. [file 1471-2105-14-68-S2.pdf]

This table describes the feature clusters derived from correlation analysis. For this purpose, the Ward clustering algorithm was used, with absolute Pearson correlation distance as metric.

Single letters stands for single amino acid frequencies according to their one-letter code, while letter pairs stands for dimer frequencies. Also, symbols α, β and $-$, stands for frequencies of alpha helices, beta sheets and coils respectively.

**Group**	**Main Feature**	**Features in the cluster**
1	Protein length	Length, Weight, G, S, T, V, DL, DS, EL, ES, GG, GS, GT, GV, KL, LD, LE, LK, LR, RL, SD, SE, SG, SS, ST, SV, TG, TS, TT, TV, VG, VS, VT, VV.
2	Negative charge / Acidic	Isoelectric point, percentage of negatively charged residues, D, E, DD, DE, ED, EE.
3	Positive charge / Basic	Hidropathicity index (GRAVY), Percentage of negatively charged residues, K, AE, AK, DK, EA, EK, EP, ET, EV, FK, GK, KA, KD, KE, KF, KG, KK, KP, KS, KT, KV, PE, PK, SK, TE, TK, VE, VK.
4	Alanine	A, AA, AG, AS, AT, AV, GA, SA, TA, VA.
5	Cysteine	C, AC, CA, CC, CD, CE, CF, CG, CH, CI, CK, CL, CN, CP, CQ, CR, CS, CT, CV, CW, CY, DC, FC, GC, HC, IC, KC, LC, MC, NC, PC, QC, RC, SC, TC, VC, WC, YC
6	Hidrophobic	F, I, L, AF, AI, AL, FA, FF, FG, FI, FL, FS, FT, FV, GF, GI, GL, IA, IF, IG, II, IL, IP, IS, IT, IV, LA, LF, LG, LI, LL, LS, LT, LV, PI, SF, SI, SL, TF, TI, TL, VF, VI, VL, $\beta\alpha$, α, β.
7	Histidine	H, AH, DH, FH, GH, HA, HD, HE, HF, HG, HH, HI, HK, HL, HN, HR, HS, HT, HV, HY, IH, KH, LH, NH, RH, SH, TH, VH, YH.
8	Asparagine / Methionine	M, N, AD, AM, AN, DA, DF, DG, DI, DM, DN, DP, DT, DV, EF, EG, EI, EM, EN, FD, FE, FM, FN, FP, FR, GD, GE, GM, GN, HM, HP, ID, IE, IK, IN, KI, KM, KN, LM, LN, MA, MD, ME, MF, MG, MH, MI, MK, ML, MM, MN, MP, MS, MT, MV, NA, ND, NE, NF, NG, NI, NK, NL, NM, NN, NP, NS, NT, NV, PD, PF, PH, PM, PN, PR, RF, RP, SM, SN, TD, TM, TN, VD, VM, VN.
9	Proline	P, AP, GP, LP, PA, PG, PL, PP, PS, PT, PV, SP, TP, VP.
10	Glutamine	Q, AQ, CM, DQ, EC, EH, EQ, FQ, GQ, HQ, IQ, KQ, LQ, MQ, NQ, PQ, QA, QD, QE, QG, QH, QI, QK, QL, QM, QN, QP, QQ, QS, QT, QV, QY, SQ, TQ, VQ.
11	Arginine	R, AR, DR, ER, FR, GR, IR, KR, MR, NR, QR, RA, RD, RE, RG, RI, RK, RM, RN, RQ, RR, RS, RT, RV, SR, TR, VR.
12	Tryptophan	W, AW, DW, EW, FW, GW, HW, IW, KW, LW, MW, NW, PW, QW, RW, SW, TW, VW, WA, WD, WE, WF, WG, WH, WI, WK, WL, WM, WN, WP, WQ, WR, WS, WT, WV, WW, WY, YM
13	Tyrosine	Y, AY, DY, EY, FY, GY, IM, IY, KY, LY, MY, NY, PY, QF, RY, SY, TY, VY, YA, YD, YE, YF, YG, YI, YK, YL, YN, YP, YQ, YR, YS, YT, YV, YW, YY.
14	Alpha helices	$-$, α, $--$, $-\alpha$ $\alpha-$, $\alpha\alpha$.
15	Beta sheets	β, $-\beta$, $\beta-$, $\beta\beta$.
