# Supplementary material for: Predictability of gene ontology slim-terms from primary structure information in Embryophyta plant proteins
Source: BMC Bioinformatics. 2013 Feb 26;14:68. doi: 10.1186/1471-2105-14-68 (PMC3660269; doi:10.1186/1471-2105-14-68)
Supplement: Additional file 3 — Detailed results for several identity thresholds. Figures depicting detailed results for each class with the full feature set, for several identity cutoffs. Left plots show sensitivity, specificity and geometric mean (green line) achieved with the five-fold cross-validation procedure, while right plots depicts boxplots for analyzing performance variation throughout the five repetitions. Left plots also depicts the performance of the BLASTP algorithm for comparison purposes (blue line). [file 1471-2105-14-68-S3.pdf]

The following figures depict detailed results of predicting each class with the full feature set, for several identity cutoffs. Left plots show sensitivity, specificity and geometric mean (green line) achieved with the five-fold cross-validation procedure, while right plots depicts boxplots for analyzing performance variation throughout the five repetitions. Left plots also depicts the performance of the BLASTP algorithm for comparison purposes (blue line).

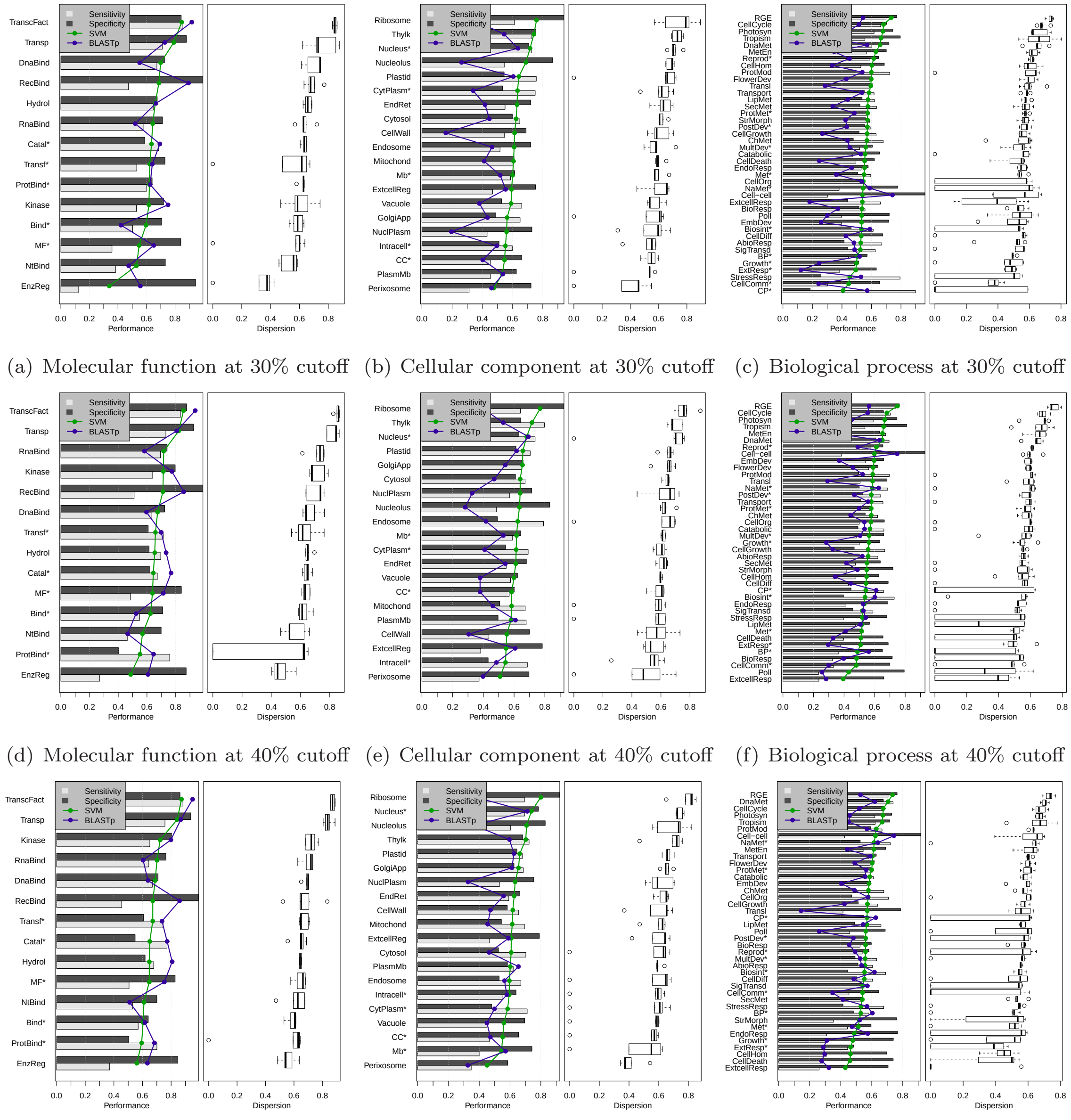

(a) Molecular function at 30% cutoff (b) Cellular component at 30% cutoff (c) Biological process at 30% cutoff

(d) Molecular function at 40% cutoff (e) Cellular component at 40% cutoff (f) Biological process at 40% cutoff

(g) Molecular function at 50% cutoff (h) Cellular component at 50% cutoff (i) Biological process at 50% cutoff

Figure 1: Detailed results for various identity percentage cutoffs

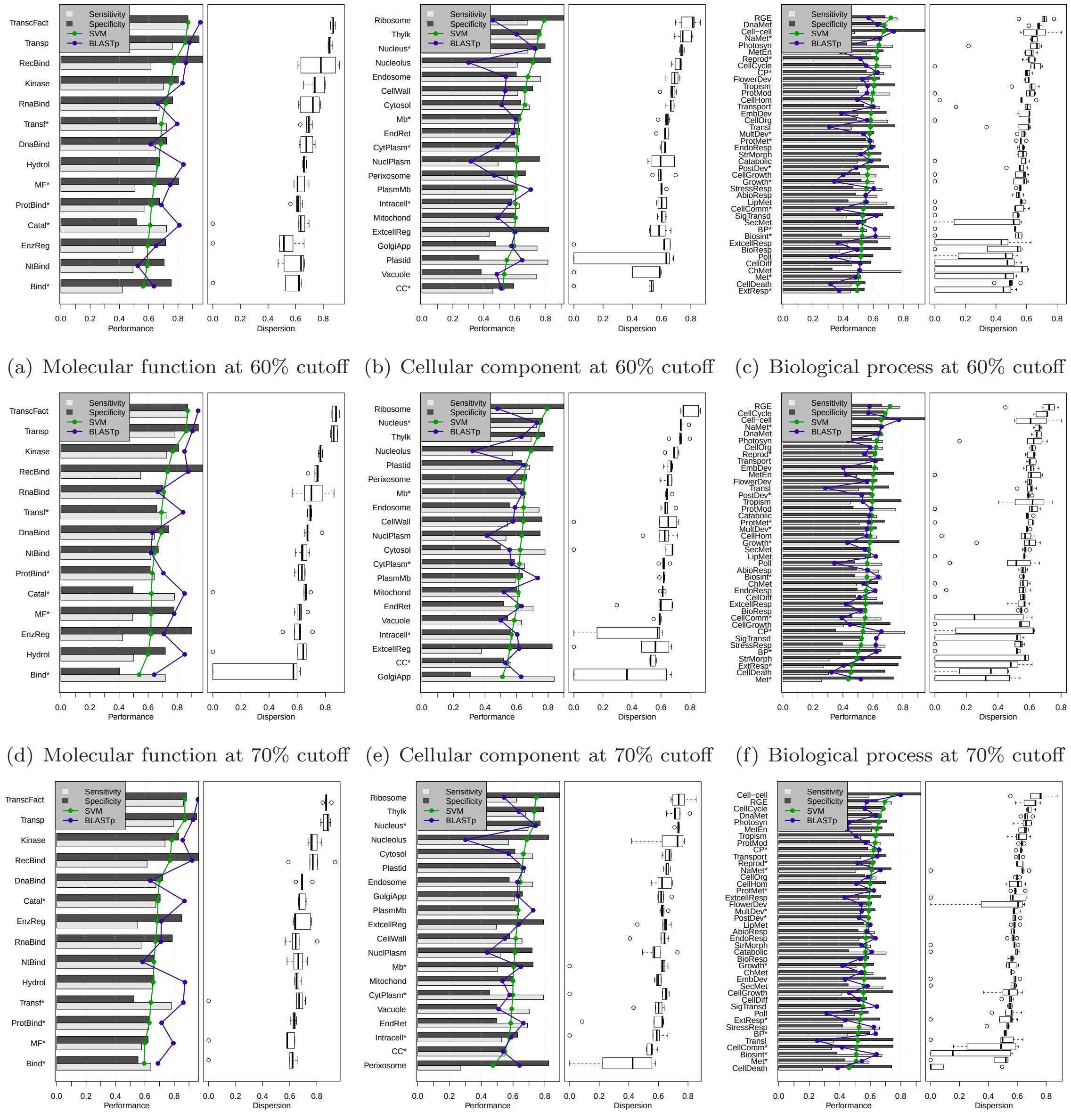

(a) Molecular function at 60% cutoff (b) Cellular component at 60% cutoff (c) Biological process at 60% cutoff

(d) Molecular function at 70% cutoff (e) Cellular component at 70% cutoff (f) Biological process at 70% cutoff

(g) Molecular function at 80% cutoff (h) Cellular component at 80% cutoff (i) Biological process at 80% cutoff

Figure 2: Detailed results for various identity percentage cutoffs
